# *Ruminococcus bovis* sp. nov., a novel species of amylolytic *Ruminococcus* isolated from the rumen of a dairy cow

**DOI:** 10.1099/ijsem.0.004924

**Published:** 2021-08-11

**Authors:** James Gaffney, Jordan Embree, Sean Gilmore, Mallory Embree

**Affiliations:** ^1^​ Native Microbials, 10255 Science Center Drive, San Diego, CA 92121, USA

**Keywords:** genome, rumen, *Ruminococcus*

## Abstract

This study describes JE7A12^T^ (=ATCC TSD-225^T^=NCTC 14479^T^), an isolate from the ruminal content of a dairy cow. Phenotypic and genotypic traits of the isolate were explored. JE7A12^T^ was found to be a strictly anaerobic, catalase-negative, oxidase-negative, coccoid bacterium that grows in chains. The API 50 CH carbon source assay detected fermentation of d-glucose, d-fructose, d-galactose, glycogen and starch. HPLC showed acetate to be the major fermentation product as a result of carbohydrate fermentation. Phylogenetic analysis of JE7A12^T^ based on 16S rRNA nucleotide sequence and amino acid sequences from the whole genome indicated a divergent lineage from the closest neighbours in the genus *Ruminococcus *. The results of 16S rRNA sequence comparison, whole genome average nucleotide identity (ANI) and DNA G+C content data indicate that JE7A12^T^ represents a novel species which we propose the name *Ruminococcus bovis* with JE7A12^T^ as the type strain.

The genus *Ruminococcus* was first described by Sijpesteijn [1] with *Ruminococcus flavefaciens* as the type strain [2]. Previously, members of the genus *Ruminococcus* have most frequently been isolated from the rumen and gastrointestinal tract of a wide variety of animals, including humans [3]. The genus is polyphyletic and divided into two groups. *Ruminococcus* group 1 includes the type strain *Ruminococcus flavefaciens *, *Ruminococcus albus *, *Ruminococcus bromii* and *Ruminococcus callidus. Ruminococcus* group 2 species have recently undergone taxonomic re-classification with many species being reassigned to different genera [4]. It is now believed that true members of the genus *Ruminococcus* are the species found in group 1 [4].

Microbial fermentation plays a prominent role in the utilization of feed by ruminants. In the rumen, bacterial fermentation is known to contribute to the stabilization of ruminal pH, increase volatile fatty acid production, reduce ammonia concentration and improve fibre digestibility [5–12]. *Ruminococci* are ubiquitous members of the human gastrointestinal and rumen microbial consortia worldwide where they play a role in the fermentation of cellulose rich feedstuffs and resistant starch [13–19]. Assessment of the global distribution of rumen microbes by Henderson *et al*. found that species of the genus *Ruminococcus* were present in all ruminants surveyed and, on average, were found to comprise 3.6 % of the total rumen bacterial community [20]. While the abundance of members of the genus *Ruminococcus* is naturally high there is evidence that their functional role is larger than the abundance would suggest. Xia *et al*. revealed that 70–80 % of the starch degrading bacteria in the barley-fed beef heifers were members of the family *Ruminococcaceae* [21]. Similarly, shotgun metagenomics approaches have demonstrated that a disproportionately high number of genes encoding hemicellulase and cellulase in the rumen can be associated with members of group 1 of the genus *Ruminococcus* [22]. Thus, characterization of novel species of the genus *Ruminococcus* has the potential to elucidate underlying microbial functionality in the rumen and the influence of members of the genus with regards to ruminant feed utilization and nutrition. The following description pertains to the isolation and classification of a novel group 1 amylolytic species, represented by strain JE7A12^T^, of the genus *Ruminococcus *.

## Isolation and ecology

JE7A12^T^ was recovered from the rumen content of a healthy, Holstein dairy cow obtained from a farm in Tulare, California, USA on a modified chopped meat broth with carbohydrates solid medium (DSMZ Medium 110) at 37 °C in an anaerobic environment (5 % H_2_, 20 % CO_2_, 75 % N_2_). The medium was modified by the removal of fat-free ground meat and casein and the addition of 30.0 g peptone, 15.0 g meat extract, 10.0 g meat peptone, 15.0 g agar and 100 ml clarified rumen fluid [23] per litre of medium. After 48 h of anaerobic incubation at 37–39 °C, JE7A12^T^ displayed off-white colonies approximately 0.1–0.3 mm in diameter on supplemented Bacto Tryptic Soy Broth (BD) with 0.4 g l-cysteine hydrochloride, 0.02 g ferric ammonium citrate, 10 µg vitamin K_1_, 2.0 mg resazurin sodium salt, 10.0 ml vitamin supplement ATCC MDVS (ATCC) and 7.0 g Gelrite (CP Kelco) per litre of medium (TSB+FAC). Gram-staining was performed as described by Beveridge [24]. Cell morphology was observed under an Accu-Scope EXC-350 light microscope using cells grown for 48 h at 37 °C on TSB+FAC. Cell size was measured using the microscopy imaging software Captavision + (Accu-Scope). Cells were Gram-stain positive, non-spore-forming and presented as small cocci (0.9–1.2 µm in diameter) (Figs S1 and S2, available in the online version of this article). The strain did not grow in the presence of oxygen and therefore is considered obligately anaerobic. Consistent with previous descriptions of the genus, JE7A12^T^ is a strictly anaerobic coccoid, commonly found in pairs and chains [3]. Although isolated from rumen content, JE7A12^T^ does not require rumen fluid for growth.

## 16S rRNA phylogeny

**Fig. 1.**
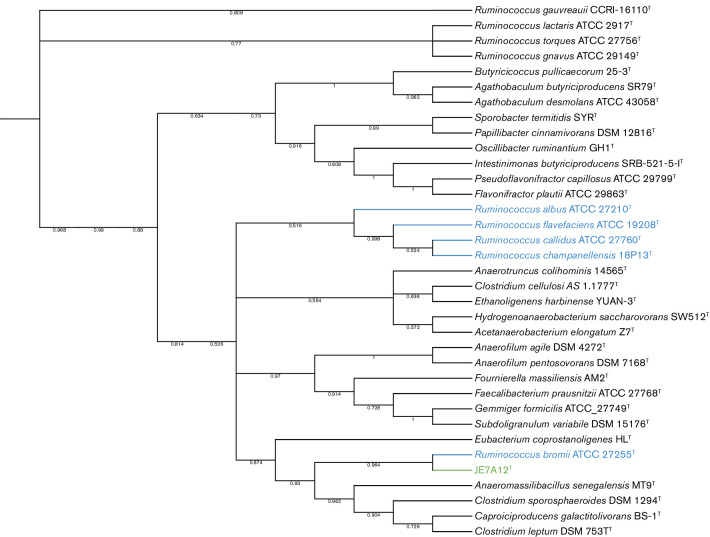
JE7A12^T^ 16S rRNA phylogenetic tree by mega X, dendrogram; JE7A12^T^ and type strains of species of the genus *Ruminococcus* as well as type strains of closely related species. The tree was reconstructed using 16S rRNA type strain sequences from members of the order *Clostridiales* in the RDP database by the neighbor-joining method based on the comparison of 1500 nt sequences. JE7A12^T^ node and label are in green type. *Ruminococcus* group 1 type strains are in blue type. Bootstrap values, resulting from 500 replications, are given at each branch point.

16S rRNA based phylogeny was computed by the neighbor-joining method using mega X [25]. JE7A12^T^ was placed in a dendrogram of all type strains of species from the order *Clostridiales* for which a full length 16S rRNA sequence was available in the RDP database [26]. The dendrogram was trimmed to include all the current members of the genus *Ruminiococcus* as well as close phylogenetic neighbours (Fig. 1).

**Fig. 2.**
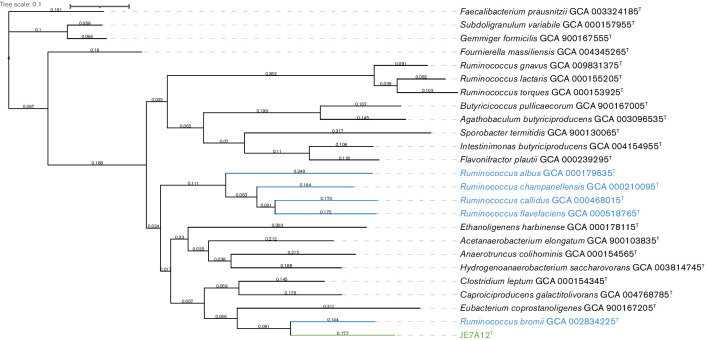
JE7A12^T^ phylogenic tree by PhyloPhlan dendrogram; JE7A12 ^T^ and type strains of species of the genus *Ruminococcus* as well as type strains of other close phylogenetic neighbours. JE7A12^T^ is indicated in green type, type strains of members of *Ruminococcus* group 1 are indicated in blue type. Branch length based on relative concatenated amino acid sequence similarity is appended to each branch. NCBI GenBank accession numbers are appended to each species label.

To confirm the results from the tree reconstructed from 16S rRNA sequences, a second phylogenetic tree was reconstructed using PhyloPhlan and a subset of 400 conserved proteins [27]. JE7A12^T^ was placed in the dendrogram generated by PhyloPhlan with type strains of species of the genus *Ruminococcus* as well as type strains of species that were close matches from the 16S rRNA phylogenetic analysis (Fig. 2). Both the 16S rRNA tree reconstructed using mega X and the tree reconstructed using PhyloPhlan placed JE7A12^T^ on a divergent branch within the *Ruminococcus* group 1 cluster. In agreement with the results of the ANI analysis, *R. bromii* was revealed to be the closest neighbour of JE7A12^T^ by both phylogenetic reconstruction methods. As previously reported, the species of *Ruminococcus* group 2 form a separate and distinct cluster.

## Genome features

DNA from a pure culture of JE7A12^T^ was extracted by a modified Sambrook phenol–chloroform extraction/purification protocol [28]. Short-read libraries for whole genome sequencing were generated using a Kapa HyperPlus kit (Roche and single-end sequenced (1×300) on a MiSeq (Illumina). In parallel, long-read libraries were generated using the SQK-RAD004 kit (Oxford Nanopore Technologies) and 1D sequenced on the MinION (R9.4 flowcell). Sequencing resulted in greater than 100× coverage by Illumina reads and 55× coverage by Oxford Nanopore. The genome was assembled by hybrid methods, utilizing both Canu [29] and Pilon [30], as described by George *et al*. [31]. The assembly resulted in the generation of a single, circular contig with a length and N50 of 2 440 231 base pairs. The DNA G+C content of the assembly is 34.6 mol%. The whole genome has been deposited at NCBI (accession number CP039381). Whole genome size and DNA G+C content were compared between JE7A12^T^ and all current members of the genus *Ruminococcus* (Table 1). At 34.6 mol%, the DNA G+C content of JE7A12^T^ should act as a differentiating characteristic for the species as it is significantly lower than those of any other member of the genus. The lowest known DNA G+C content for other members of the genus is 39 mol% for strains of *R. flavefaciens* and *R. bromii* [3].

**Table 1.** Characteristics of JE7A12^T^ compared with members of the genus *Ruminococcus
* Fermentation data for *R. champanellensis* and *R. gauvereauii* were taken from Chassard *et al.* [15] and Domingo *et al.* [42], respectively. Fermentation data for all other species were taken from Ezaki [3]. +, Positive; −, negative; sd, strain dependent; w, weak reaction; nd., no data; A, acetate; F, formate; S, succinate; *, average based on all assemblies in the NCBI database.

Characteristic	JE7A12^T^	* R. albus *	* R. bromii *	* R. callidus *	* R. champanellensis *	* R. flavefaciens *	* R. gauvreauii *	* R. gnavus *	* R. lactaris *	* R. torques *
Genomic DNA G+C content (mol%)	34.6	44.7*	41.0*	49.1*	53.3*	46.9*	47.6*	42.7*	42.7*	41.7
Genome size (Mbp)	2.44	3.71*	2.15*	3.10*	2.54*	2.70*	4.10*	3.50*	2.73*	3.00^*^
Major fermentation product(s)	A	A, F	A	A, S	A, S	A, F, S	A	A, F	A, F	A, L
Fermentation of:										
Arabinose	–	–	–	–	nd	–	nd	+	–	–
Cellobiose	–	+	–	+	+	+	–	–	–	–
Glucose	+	+	+	+	–	–	nd	+	+	+
Lactose	–	+	–	+	–	+	–	–	+	+
Mannose	–	+	w/–	–	–	–	nd	–	w/–	w/–
Maltose	+	–	+	+	–	–	nd	+	sd	w
Mannitol	–	–	–	–	nd	–	nd	–	+	–
Raffinose	–	–	–	+	–	–	–	+	–	–

The full length 16S rRNA sequence of JE7A12^T^ was extracted from the whole genome sequence. The authenticity of the assembled 16S rRNA sequence was confirmed by comparison with a 16S rRNA amplicon sequence obtained using the 27F and 1492R primers and previously described methods [32]. The full length 16S rRNA sequence was subsequently compared with entries in the NCBI database by blast. Excluding species without validly published names, the closest neighbours to JE7A12^T^ based on 16S rRNA sequence similarity are *Ruminococcus bromii* (93.3 %), *Clostridium leptum* (91.2 %) and *Caproiciproducens galactitolivorans* (89.2 %).

To further investigate taxonomic identity, whole genome average nucleotide identity (ANI) was compared between JE7A12^T^ and type strains for all current species of the genus *Ruminococcus* [33]. Additionally, type strains of *Clostridium leptum* and *Caproiciproducens galactitolivorans* were included in the ANI analysis due to their close 16S rRNA similarity. Due to bias in ANI algorithms, the ANI of JE7A12^T^ was evaluated utilizing both MUMmer and blast algorithms [34–36] (Tables 2 and 3).There were no matches at the suggested 95 % cutoff for defining a species [33, 34, 37]. The best match by blast was to *Ruminococcus bromii *. However, the two species are genetically distant as their genomes share 72.7 % sequence similarity but at only 20.1 % coverage of the genome (Table 3). MUMmer offered higher sequence similarity matches than blast, with sequence alignment values between 81.7 and 93.9 % for all species. However, these matches exhibited very low genome coverage (Table 2). The only species which demonstrates greater than 0.3 % genome coverage was *Ruminococcus bromii *. Whole genome nucleotide dissimilarity is a strong differentiator of JE7A12^T^ from the other taxa of the genus.

**Table 2.** Average nucleotide identity by MUMmer ANI using MUMmer between JE7A12^T^ and type strains of species of the genus *Ruminococcus* as well as type trains of closely related species as determined by 16S rRNA sequence alignment.

Genus species (Genbank accession number)	ANI (%)	Coverage (%)
* Ruminococcus gauvreauii* CCRI-16110^T^ (GCA_000425525)	**93.7**	**0.11**
* Caproiciproducens galactitolivorans* BS-1T^T^ (GCA_004768785)	**92.5**	**0.31**
* Clostridium leptum* VPI T7-24-1^T^ (GCA_000154345)	**89.8**	**0.16**
* Ruminococcus champanellensis* 18 P13^T^ (GCA_000210095)	**88.8**	**0.24**
* Ruminococcus callidus* VPI57-31^T^ (GCA_000468015)	**87.8**	**0.20**
* Ruminococcus flavefaciens* ATCC 19208^T^ (GCA_000518765)	**86.0**	**0.21**
* Ruminococcus albus* ATCC 27210^T^ (GCA_000179635)	**85.8**	**0.14**
* Ruminococcus bromii* VPI 6883^T^ (GCA_002834225)	**84.5**	**1.66**
* Ruminococcus gnavus* VPI C7-9^T^ (GCA_009831375)	**82.3**	**0.13**
* Ruminococcus torques* VPI B2-51^T^ (GCA_000153925)	**82.2**	**0.18**
* Ruminococcus lactaris* VPI X6-29^T^ (GCA_000155205)	**81.7**	**0.21**

**Table 3.** Average nucleotide identity by blast ANI using blast between JE7A12^T^ type strains of species of the genus *Ruminococcus* as well as type species of closely related species as determined by 16S rRNA sequence alignment.

Genus species (Genbank accession number)	ANI (%)	Coverage (%)
* Ruminococcus bromii* VPI 6883^T^ (GCA_002834225)	**72.8**	**20.2**
* Ruminococcus torques* VPI B2-51^T^ (GCA_000153925)	**71.9**	**3.32**
* Ruminococcus albus* ATCC 27210^T^ (GCA_000179635)	**71.6**	**2.76**
* Ruminococcus flavefaciens* ATCC 19208^T^ (GCA_000518765)	**70.9**	**3.28**
* Ruminococcus gnavus* VPI C7-9^T^ (GCA_009831375)	**70.9**	**2.54**
* Ruminococcus lactaris* VPI X6-29^T^ (GCA_000155205)	**70.8**	**3.69**
* Caproiciproducens galactitolivorans* BS-1T^T^ (GCA_004768785)	**70.1**	**5.48**
* Ruminococcus champanellensis* 18 P13^T^ (GCA_000210095)	**70.1**	**2.88**
* Ruminococcus callidus* VPI57-31^T^ (GCA_000468015)	**70.0**	**3.08**
* Clostridium leptum* VPI T7-24-1^T^ (GCA_000154345)	**69.7**	**3.93**
* Ruminococcus gauvreauii* CCRI-16110^T^ (GCA_000425525)	**69.3**	**1.38**

## Physiology and chemotaxonomy

Catalase and oxidase activities of JE7A12^T^ were determined using a 3 % (v/v) hydrogen peroxide solution and 1.2 % tetra-methyl-*p*-phenylenediamine dihydrochloride solution, respectively. Growth temperature ranges were determined in TSB+FAC medium at 25, 30, 37, 40 and 50 °C. Optimal growth was observed at 37 and 40 °C, reduced growth at 30 °C and no growth at 25 and 50°C incubation temperature. No motility was observed. Growth in the presence of salt was studied by supplementing TSB+FAC liquid medium with NaCl (0.5–4.5 % w/v in 0.5 % increments). Hungate tubes were incubated at 37 °C for 72 h and monitored for growth. JE7A12^T^ was capable of growing in salt concentrations of up to 2.5 %. Tolerance to pH (5.0–9.0) was tested on TSB+FAC with pH tested in increments of 0.5 pH units. Hungate tubes were incubated at 37 °C for 72 h and monitored for growth. The optimal pH for growth was pH 7.0–7.5 with reduced growth at pH 6.0–6.5. No growth was observed at pH 8.0–9.0 and pH 5.0–5.5 on TSB+FAC.

Carbohydrate fermentation of JE7A12^T^ was qualitatively measured using the API 50CH carbon panel (BioMérieux). JE7A12^T^ cells were grown to late exponential phase and recovered by centrifugation at 3000 ***g*** for 10 min. Cells were resuspended and 0.017 % (w/v bromocresol purple added as a pH indicator for acidification of carbohydrates [38]. JE7A12^T^ fermented d-galactose, d-glucose, d-fructose, maltose, glycogen, aesculin/ferric citrate and starch. No fermentation of glycerol, erythritol, d-arabinose, l-arabinose, d-ribose, d-xylose, l-xylose, methyl β-d-xylopyranoside, d-cellobiose, d- adonitol, d-lactose, d-saccharose, d-trehalose, d-melibiose, d-mannose, l-arabitol, l-sorbose, l-rhamnose, dulcitol, inositol, d-mannitol, d-sorbitol, methyl d-mannopyranoside, methyl d-glucopyranoside, *N*-acetyl glucosamine, amygdalin, arbutin, melezitose, raffinose, xylitol,inulin, salicin, gentiobiose, turanose, d-lyxose, d-tagatose, d-fucose, l-fucose, d-arabitol, potassium gluconate, potassium 2-ketogluconate and potassium 5-ketogluconate was observed (Table S1).

A comparison of carbon source fermentation between JE7A12^T^ and all current species of the genus *Ruminococcus* can be found in Table 1. Similarly to *R. bromii *, JE7A12^T^ shows narrow specialization with regards to carbohydrate fermentation, while other members of the genus *Ruminococcus* generally ferment a wider range of carbohydrates [39]. Specifically, *R. bromii* has been reported to ferment most of the same carbon sources as JE7A12^T^, including galactose, glucose, fructose, maltose, glycogen and starch [3, 40]. Despite the similarities between the species, strains of *R. bromii* derived from the bovine rumen are not known to ferment fructose or galactose and are rarely able to ferment glucose. Utilization of these carbon sources have more commonly been observed in human derived *R. bromii* [41]. Therefore, fermentation of glucose, fructose and galactose could act to differentiate JE7A12^T^ from ruminally derived *Ruminococcus bromii *.

Metabolite production was measured using a Waters Acquity UPLC Q System with RI detector. The column used was a Phenomenex 00 H-0138-K0 Rezex ROA Organic Acid H+ (8 %) operated at 60 °C. The mobile phase was 0.001625 M H_2_SO_4_ at 0.5 ml min^−1^. Pure standards of acetate, ethanol, glycerol, lactate, butyrate, butanol, propionate, succinate and pyruvate were used for calibration at varying concentrations. JE7A12^T^ produces acetate as a major fermentation product as well as ethanol and glycerol as minor products. No lactate, butyrate, butanol, propionate, succinate or pyruvate is produced. A comparison of metabolite production between JE7A12^T^ and all current species of the genus *Ruminococcus* can be found in Table 1. The fermentation profile of JE7A12^T^ most closely resembles that of *R. bromii* and *R. gauvreauii* which are the only species in the genus that produce acetate, and only acetate, as a major metabolic product. While the other members of the genus produce acetate, they also produce high levels of succinate, lactate and formate.

## Description of *Ruminococcus bovis* Sp. Nov.

*Ruminococcus bovis* (bo'vis. L. gen. n. *bovis* of the cow)

*Ruminococcus bovis* is an obligately anaerobic, catalase-negative and oxidase-negative bacterium. It is Gram-stain-positive and forms chains of small cocci when cultured in liquid medium. When cultured on TSB+FAC solid medium, it forms small, slightly opaque, off-white, circular colonies with even margins. Fermentation of d-galactose, d-glucose, d-fructose, maltose, glycogen, aesculin/ferric citrate and starch is indicated by API CH50. The major fermentation product is acetate, with ethanol and glycerol as minor products. No lactate, butyrate, butanol, propionate, succinate or pyruvate is produced.

The type strain is JE7A12^T^ (=ATCC TSD-225^T^=NCTC 14479^T^) and was originally isolated from rumen content of a healthy, Holstein cow from Tulare, California, USA. The genomic DNA G+C content of the type strain is 34.6 mol%.

## Supplementary Data

Supplementary material 1
