# Supplementary material for: Ruminococcus bovis sp. nov., a novel species of amylolytic Ruminococcus isolated from the rumen of a dairy cow
Source: Int J Syst Evol Microbiol. 2021 Aug 11;71(8):004924. doi: 10.1099/ijsem.0.004924 (PMC8513621; doi:10.1099/ijsem.0.004924)
Supplement: Supplementary material 1 [file ijsem-71-4924-s001.pdf]

Supplementary Figure 1. Methylene Blue Stain

Methylene blue stain of JE7A12^T after 48 hours of incubation viewed at 1000x magnification.

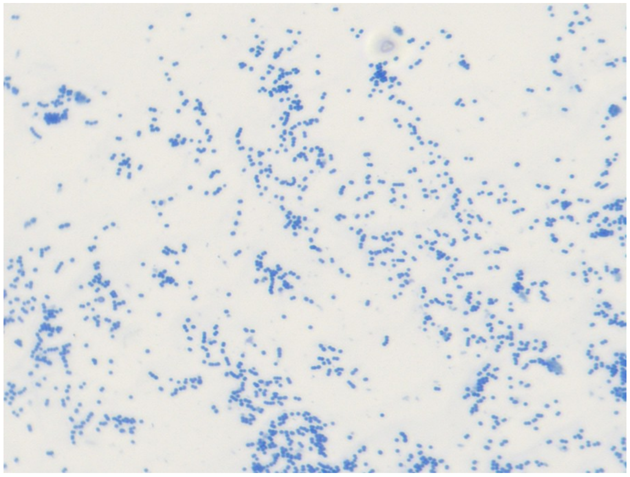

Supplementary Figure 2. Gram Stain

Gram stain of JE7A12^T after 48 hours of incubation viewed at 1000x magnification.

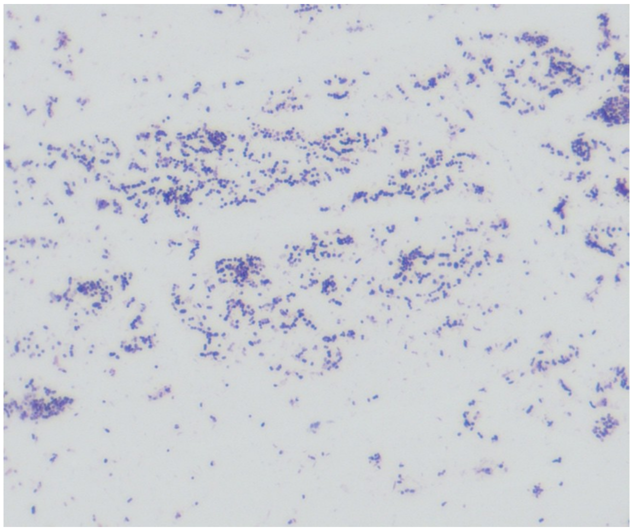

Supplementary Table 1. JE7A12^T API 50CH carbon panel

Carbon Source	JE7A12^T	Carbon Source	JE7A12^T	Carbon Source	Growth
Control	-	Inositol	-	D-Melezitose	-

**Glycerol**	-	**D-Mannitol**	-	**D-Raffinose**	-
**Erythritol**	-	**D-Sorbitol**	-	**Starch**	+
**D-Arabinose**	-	**Methyl-aD Mannopyranoside**	-	**Glycogen**	+
**L-Arabinose**	-	**Methyl-aD Glucopyranoside**	-	**Xylitol**	-
**D-Ribose**	-	**N-AcetylGlucosamine**	-	**Gentiobiose**	-
**D-Xylose**	-	**Amygdalin**	-	**D-Turanose**	-
**L-Xylose**	-	**Arbutin**	-	**D-Lyxose**	-
**D-Adonitol**	-	**Esculin/Ferric Citrate**	+	**D-Tagatose**	-
**Methyl-BD xylopyranoside**	-	**Salicin**	-	**D-Fucose**	-
**D-Galactose**	+	**D-Cellobiose**	-	**L-Fucose**	-
**D-Glucose**	+	**D-Maltose**	+	**D-Arabitol**	-
**D-Fructose**	+	**D-Lactose**	-	**L-Arabitol**	-
**D-Mannose**	-	**D-Melibiose**	-	**Potassium Gluconate**	-

**L-Sorbose**	-	**D-Saccharose**	-	**Potassium 2-KetoGluconate**	-
**L-Rhamnose**	-	**D-Trehalose**	-	**Potassium 5-KetoGluconate**	-
**Dulcitol**	-	**Inulin**	-		
